# Resolving diverse protein–DNA footprints from exonuclease-based ChIP experiments

**DOI:** 10.1093/bioinformatics/btab274

**Published:** 2021-07-12

**Authors:** Anushua Biswas, Leelavati Narlikar

**Affiliations:** Department of Chemical Engineering, CSIR-National Chemical Laboratory, Pune 411008, India; Academy of Scientific and Innovative Research, Ghaziabad 201002, India; Department of Chemical Engineering, CSIR-National Chemical Laboratory, Pune 411008, India; Academy of Scientific and Innovative Research, Ghaziabad 201002, India

## Abstract

**Motivation:**

High-throughput chromatin immunoprecipitation (ChIP) sequencing-based assays capture genomic regions associated with the profiled transcription factor (TF). ChIP-exo is a modified protocol, which uses lambda exonuclease to digest DNA close to the TF-DNA complex, in order to improve on the positional resolution of the TF-DNA contact. Because the digestion occurs in the 5′–3′ orientation, the protocol produces directional footprints close to the complex, on both sides of the double stranded DNA. Like all ChIP-based methods, ChIP-exo reports a mixture of different regions associated with the TF: those bound directly to the TF as well as via intermediaries. However, the distribution of footprints are likely to be indicative of the complex forming at the DNA.

**Results:**

We present ExoDiversity, which uses a model-based framework to learn a joint distribution over footprints and motifs, thus resolving the mixture of ChIP-exo footprints into diverse binding modes. It uses no prior motif or TF information and automatically learns the number of different modes from the data. We show its application on a wide range of TFs and organisms/cell-types. Because its goal is to explain the complete set of reported regions, it is able to identify co-factor TF motifs that appear in a small fraction of the dataset. Further, ExoDiversity discovers small nucleotide variations within and outside canonical motifs, which co-occur with variations in footprints, suggesting that the TF-DNA structural configuration at those regions is likely to be different. Finally, we show that detected modes have specific DNA shape features and conservation signals, giving insights into the structure and function of the putative TF-DNA complexes.

**Availability and implementation:**

The code for ExoDiversity is available on https://github.com/NarlikarLab/exoDIVERSITY.

**Supplementary information:**

Supplementary data are available at *Bioinformatics* online.

## 1 Introduction

Transcriptional regulation is governed by interactions between proteins called transcription factors (TFs) and their corresponding DNA binding sites. TFs may bind DNA directly or through complexes with other proteins, which make contact with DNA. Identifying these genomic binding sites is critical to understand their role in gene-regulation. High-throughput chromatin immunoprecipitation (ChIP) is the method of choice for locating DNA regions associated with a TF or protein of interest (POI) (Furey, 2012). Here, after protein-protein and protein-DNA interactions are cross-linked in vivo, fragmented chromatin associated with the POI is immunoprecipitated. The identity of the fragments is usually discerned using sequencing (ChIP-seq). After mapping the reads back to the genome, one typically gets genomic regions of length between 100 and 1000 bp: the short 5–20 bp TF-DNA binding site (direct or otherwise) could be anywhere within that region.

To improve the positional resolution of these binding sites, a variation of the method was developed, which uses the lambda exonuclease after the immunoprecipitation step (Rhee and Pugh, 2011). The enzyme digests chromatin to the point where the cross-linked protein-DNA complex hinders its activity. Specifically, it degrades single strand of unbounded DNA in the 5′–3′ direction, leaving the DNA sequences 3′ of the cross-link intact. After sequencing and mapping the reads, one therefore gets directional footprints close to the complex, on both sides. Alignment of these footprints is expected to reveal the DNA contacts made by the POI. However, the footprints are not the same at all locations and depend on multiple factors: (i) the actual protein occupying the DNA, which may be either the POI or a protein with which it complexes/co-occurs, (ii) the nucleotide sequence, since a TF typically can bind a set of distinct nucleotide sequences, at times with varying affinities and (iii) the precise location of the cross-link within the protein-DNA complex, which obstructs the exonuclease activity resulting in the eventual distribution of reads (Rhee and Pugh, 2011).

One therefore needs to categorize these footprints in order to understand the various protein-DNA complexes that may be forming at the reported regions. Two approaches have been pursued for this. The first scans the reported regions with pre-established DNA motifs of interest and extracts putative TF binding sites. The footprints are analyzed separately at these regions to gain insights into potential complexes forming there (Starick *et al.*, 2015). The second approach, developed by Yamada *et al.* (2019), aims to learn footprints jointly from the DNA sequence and reads. Their method ChExMix first initializes potential binding events into subtypes, based either on de novo motifs or on diverse read distributions detected from the top (500–1000) most enriched regions, and then refines them using expectation maximization in subsequent iterations over the complete set. We propose a new approach ExoDiversity that does not rely on motifs to be identified a priori and similar to ChExMix divides the ChIP-exo binding events into different binding modes based on both the read distributions and the sequence motifs. However, unlike ChExMix, ExoDiversity is built on a model-based Bayesian framework that uses both sources of information simultaneously on the complete set of regions. We show that ExoDiversity resolves read footprints to a single nucleotide level, which correlate with single nucleotide differences in the sequence motif. The discovered modes provide insights into how the DNA shape might be influencing protein-DNA binding as well as exonuclease digestion activity at those complexes.

## 2 Approach

The input to ExoDiversity are the bound regions and the corresponding mapped reads in both directions, control subtracted if the control experiment is available. ExoDiversity partitions the regions into diverse binding modes, each categorized by a DNA motif and a corresponding read footprint, all of which are learned simultaneously. Multiple models with different numbers of modes are learned and the best model is selected using the Bayesian information criterion (BIC).

## 3 Materials and methods

### 3.1 Model description

From the experiment, we have the following:

- *n* reported DNA sequences $X=X_{1},X_{2},\ldots ,X_{n}$. Here $X_{i,j}$ is one of {A, C, G, T, N}; $1\leq j\leq L_{i}$ where *L_i_* is the length of $X_{i}$.
- *n* vectors $R^{+}=R_{1}{}^{+},R_{2}{}^{+},\ldots ,R_{n}{}^{+}$, which store read counts corresponding to ***X*** on the positive strand. $R_{i,j}^{+}$ denotes the number of reads whose 5′ end maps to the genomic location corresponding to $X_{i,j}$ on the positive strand. These counts are discretized to binary values based on the median read count or a user-defined threshold.
- *n* vectors $R^{-}$ similar to above but for the negative strand.

**Fig. 1.**
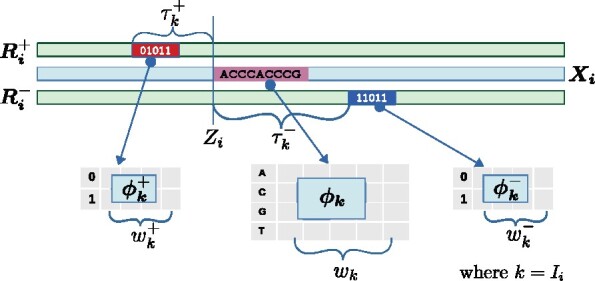
The *i*th datapoint {DNA sequence $X_{i}$, positive reads $R_{i}{}^{+}$, negative reads $R_{i}{}^{-}$}, belonging to mode *k* is shown. The red (blue) box of width $w_{k}^{+}(w_{k}^{-})$ denotes the positive (negative) read window, which is modeled using Bernoulli probabilities $\phi_{k}{}^{+}(\phi_{k}{}^{-})$. The 1 and 0 indicate the presence and absence of reads at those positions. The pink box is the DNA site at position *Z_i_*, modeled using PWM parameters $\phi_{k}$. The positive (negative) read window is at a fixed distance $\tau_{k}^{+}(\tau_{k}^{-})$ from *Z_i_*. Only $X_{i},\;R_{i}{}^{+}$ and $R_{i}{}^{-}$ are known; the rest are unknown and learned from the data

A binding mode is characterized with a joint probability distribution over the DNA motif and the read footprint. The motif is modelled as a product of categorical distributions over the four nucleotides [the standard position weight matrix, or PWM (Staden, 1984)] and the footprint is modeled as a product of Bernoulli distributions over the positive and negative reads at two small ‘read windows’, respectively, near the motif (Fig. 1). Since the lambda exonuclease digested reads are concentrated at a few bases near the binding region, we consider a read window size of five, but this can be changed by the user (Discussion). The position of these two read windows with respect to the motif, the motif locations within ***X***, the motif probabilities, as well as the Bernoulli probabilities are all unknown a priori. The goal is to partition the dataset into *m* binding modes i.e. learn parameters $\theta_{m}$ of a model $M_{m}$, which has six components {Z,I,w,ϕ,τ,γ}:

- Z_i_: position of the motif in $X_{i}$
- I_i_: binding mode of $X_{i}$; $1\leq I_{i}\leq m$
- w_k_: width of the PWM motif in mode k; 1≤k≤m
-
$w_{k}^{+}$ : width of the read window defining the positive strand footprint in mode k; here it is set to 5, but can be changed by the user
-
$w_{k}^{-}$ : as above, but for the negative strand
-
$\phi_{k}$ : PWM values for motif of mode k. $\phi_{k,a}(b)$ is the probability of finding nucleotide b at position a in PWM of mode k; $1\leq a\leq w_{k}$
-
$\phi_{k}{}^{+}$ : Bernoulli probabilities of finding a read on the positive strand for mode k. $\phi_{k,a}^{+}(1)$ is the probability of having a positive strand read at position a within the read window of mode k, while $\phi_{k,a}^{+}(0)=1-\phi_{k,a}^{+}(1)$ is the probability of finding no read there; $1\leq a\leq w_{k}^{+}$
-
$\phi_{k}{}^{-}$ : Bernoulli probabilities for negative strand reads in mode k similar to above
-
$\phi_{0}$ : parameters of the background distribution over the DNA sequences (2nd order Markov model learned directly from X)
-
$\phi_{0}{}^{+}$ and $\phi_{0}{}^{-}$: parameters of the background strand read distribution (0${}^{\text{th}}$ order Markov model learned directly from $R^{+}$ and $R^{-}$, respectively)
-
$\tau_{k}^{+}$ : distance of the start of positive strand read window from the start of motif in mode k (positive read window offset)
-
$\tau_{k}^{-}$ : distance of the start of the motif from the start of the negative strand read window in mode k (negative read window offset)
-
γ : Categorical distribution over m modes. γ_k_ is the probability of a sequence containing the binding mode k.

Assuming that the DNA and the read data are independent conditional on the mode information, the likelihood of datapoint *i* becomes:

(1) $$\begin{array}{c} P(X_{i},R_{i}{}^{+},R_{i}{}^{-}|\theta_{m},M_{m})=P(X_{i}|\theta_{m},M_{m})\\ \;\;\;\;\;\;\;\;\;\;\;\times P(R_{i}{}^{+}|\theta_{m},M_{m})\times P(R_{i}{}^{-}|\theta_{m},M_{m}) \end{array}$$

The first term in Eq. (1) is the likelihood of $X_{i}$ modelled with a motif of mode *I_i_* at position *Z_i_* and background nucleotide distribution elsewhere:

(2) $$\begin{array}{c} P(X_{i}|\theta_{m},M_{m})=P(X_{i,1},X_{i,2},\ldots ,X_{i,Z_{i}-1}|\phi_{0})\\ \;\;\;\;\times \prod_{a=1}^{w_{I_{i}}}\phi_{I_{i},a}(X_{i,Z_{i}+a-1})\\ \;\;\;\;\;\;\;\times P(X_{i,Z_{i}+w_{I_{i}}},\ldots ,X_{i,L_{i}}|\phi_{0})\\ \;\;\;\;\;\;\;\;\propto \frac{\prod_{a=1}^{w_{I_{i}}}\phi_{I_{i},a}(X_{i,Z_{i}+a-1})}{P(X_{i,Z_{i}},\ldots ,X_{i,Z_{i}+w_{I_{i}-1}}|\phi_{0})} \end{array}$$

which we get by normalizing by the background probability. Similarly the second (third) term of Eq. (1) refers to the positive (negative) strand reads, modelled with the window probabilities near the motif and background at other places.

(3) $$\begin{array}{c} P(R_{i}^{s}|\theta_{m},M_{m})=P(R_{i,1}^{s},\ldots ,R_{i,Z_{i}^{s}-1}^{s}|\phi_{0}{}^{s})\\ \;\;\;\;\;\;\;\times \prod_{a=1}^{w_{I_{i}}^{s}}\phi_{I_{i},a}^{s}(R_{i,Z_{i}^{s}+a-1})\\ \;\;\;\;\;\;\;\times P(R_{i,Z_{i}^{s}+w_{I_{i}}^{s}}^{s},\ldots ,R_{i,L_{i}}^{s}|\phi_{0}{}^{s})\\ \;\;\;\;\;\;\;\propto \frac{\prod_{a=1}^{w_{I_{i}}^{s}}\phi_{I_{i},a}^{s}(R_{i,Z_{i}^{s}+a-1}^{s})}{P(R_{i,Z_{i}^{s}},\ldots ,R_{i,Z_{i}^{s}+w_{I_{i}}^{s}-1}^{s}|\phi_{0}{}^{s})} \end{array}$$

where s∈{+,−} and the position of the read window $Z_{i}^{s}=Z_{i}+\tau_{I_{i}}^{s}$.

The full likelihood of the dataset is:

$$P(X,R^{+},R^{-}|\theta_{m},M_{m})=\prod_{i=1}^{n}P(X_{i},R_{i}{}^{+},R_{i}{}^{-}|\theta_{m},M_{m})$$

We note that the motifs can be on either strand and the footprints need not be symmetric. To handle this, the original data is appended with its reverse complement, taking care to reverse the read counts appropriately.

### 3.2 Model learning

The goal is to learn the parameters of the model $M_{m}$ to maximize the posterior distribution:

(4) $$\underset{\theta_{m}}{\text{argmax}}P(\theta_{m}|X,R^{+},R^{-},M_{m})$$

$\theta_{m}$ is a high dimensional vector. We therefore use collapsed Gibbs sampling targeting a marginalized posterior, as has been done previously with motif discovery (Liu, 1994; Mitra *et al.*, 2018). Here, we integrate out (i.e. marginalize over) $\phi_{k},\phi_{k}{}^{+},\phi_{k}{}^{-}$ and γ, while iteratively sampling the other parameters: locations of motifs (*Z_i_*) & mode of binding (*I_i_*) for each datapoint *i* and read window offsets ($\tau_{k}^{+},\tau_{k}^{-}$) and width of motifs (*w_k_*), for each binding mode *k*. We assume conjugate Dirichlet/Beta priors for this purpose. The derivation of all the sampling expressions is detailed in Supplementary Methods.

### 3.3 Model selection

We score each sample from the Gibbs sampler using the posterior distribution of eq. (4) and stop sampling when the value does not increase for 5×n iterations. We further do a hill climbing routine starting from highest scoring instance (Mitra *et al.*, 2018) and report the final instance of the read distributions and the motifs as the MAP estimate of Eq. (4). As we do not know the value of *m* before hand, we learn models with different values of *m*. We calculate the Bayesian Information Criterion (BIC) score for each model, which is a function of the posterior score and the number of free parameters (Murphy, 2021). The model with the minimum BIC score is reported as the best model.

(5) $${\text{BIC}}_{M_{m}}=K\ln(n)-2\ln(\hat{P})$$

*K* is the number of free parameters, which for model $M_{m}$ include the three parameters for every column of each PWM, one parameter for every position of each read window, and two parameters for the read position offsets per mode, while $\hat{P}$ is the posterior score of the model at the MAP estimate.

$$K=\sum_{i=1}^{m}\left(3w_{i}+w_{i}^{+}+w_{i}^{-}+2\right)$$

### 3.4 Using models to predict

Given a new datapoint $D=\{X,R^{+},R^{-}\}$, where DNA sequence ***X*** is of length *L* with associated read counts $R^{+}$ and $R^{-}$, we can calculate the probability of it belonging to mode *k* of model $M_{m}$, by marginalizing over all possible motif positions *Z*:

(6) $$P(I=k|D,\theta_{m},M_{m})\propto \gamma_{k}\sum_{j=1}^{L}P(D,Z=j|\theta_{m},M_{m})$$

where

(7) $$\begin{array}{c} P(D,Z=j|\theta_{m},M_{m})=\frac{1}{L}P(X|Z=j,\phi_{k})\\ \times P(R^{+}|Z=j,\phi_{k}^{+},\tau_{k}^{+})\times P(R^{-}|Z=j,\phi_{k}^{-},\tau_{k}^{-}) \end{array}$$

The three individual probability terms are computed using Equations 2 and 3. In Figure 3B, after the model is learned, the confusion matrix entry (*u*, *v*) is computed as the average probability of assigning a datapoint of mode *u* to mode *v*:

$$\frac{1}{\text{number}\;\text{of}\;\text{regions}\;\text{in}\;\text{mode}\;u}\sum_{\overset{i=1}{I_{i}=u}}^{n}P(I_{i}=v|D_{i},\theta_{m},M_{m})$$

In cases where only motif or only reads are used, the corresponding terms are dropped from eq. (7).

We can similarly compute the probability of the datapoint given model $M_{m}$ by marginalizing over *I* and *Z*:

(8) $$P(D|\theta_{m},M_{m})=\sum_{k=1}^{m}\gamma_{k}\sum_{j=1}^{L}P(D,Z=j|\theta_{m},M_{m})$$

assuming the position *Z* of the motif to be equally likely to appear anywhere within ***X***.

Given two models $M_{a}$ and $M_{b}$, we can compute the log odds score for the datapoint:

(9) $$S(D)=\text{log}\;\frac{P(D|\theta_{a},M_{a})}{P(D|\theta_{b},M_{b})},$$

which is used for classifying ***D***. Eq. (6) is used in Supplementary Figure S2, while eq. (9) is used in Figure 4.

### 3.5 Datasets

#### 3.5.1 In silico mixed data

MultiGPS 0.74 (Mahony *et al.*, 2014) was run on BAM files from CTCF and FoxA1 experiments in the MCF7 cell-line (GSE110502) separately with the corresponding control file to obtain the summits for each TF. Regions of length 240 bp around the summits were extracted and 5000 regions were randomly chosen from each dataset, ensuring that they did not contain repetitive DNA or overlapped with each other. The 10 000 regions were mixed, and the BAM files from both experiments were merged. The regions file and the mixed & control BAM files were all given to ChExMix to run with its default parameters. The mixed reads were control subtracted after scaling and along with the peak regions given to ExoDiversity to run with its default parameters. ExoDiversity was run with default parameters on all datasets mentioned in the subsequent sections as well, except in the case of CTCF, where the initial motif width was set to 40.

#### 3.5.2 Full datasets

ChIP-nexus data for Sox2, Oct4, Klf4 and Nanog are in mouse ESCs (Avsec *et al.*, 2021). The bigWig files containing the read counts and the peak files were taken from the GEO database (GSE137193), which used the MACS2 peak finder (Feng *et al.*, 2012). ChIP-nexus reads data for Twist in Drosophila (He *et al.*, 2015) was taken from GSE55306. MultiGPS peaks for the individual sets of FoxA1 and CTCF were used as input without mixing when the full individual sets were analysed by ExoDiversity. MultiGPS peaks for ER*α* (Yamada *et al.*, 2019) along with its read counts were taken from GSE110502. Reads for Glucocorticoid Receptor(GR) (Starick *et al.*, 2015) in U2OS cell lines was taken from EBI ArrayExpress (E-MTAB-2955). ChIP-seq peak file (E-MTAB-2956) was used as the input peak regions for ExoDiversity. For all datasets, 240 bp around the summits were extracted, repeats were masked, and only regions with at least 100 bp of non-repetitive DNA were used as input.

#### 3.5.3 Other data

DNA shape data is from GBShape website (Chiu *et al.*, 2015). Placental phastCons scores and gene annotations from refGene file were obtained from the UCSC Genome Browser (Karolchik *et al.*, 2004).

## 4 Results

### 4.1 In silico mixed data

**Fig. 2.**
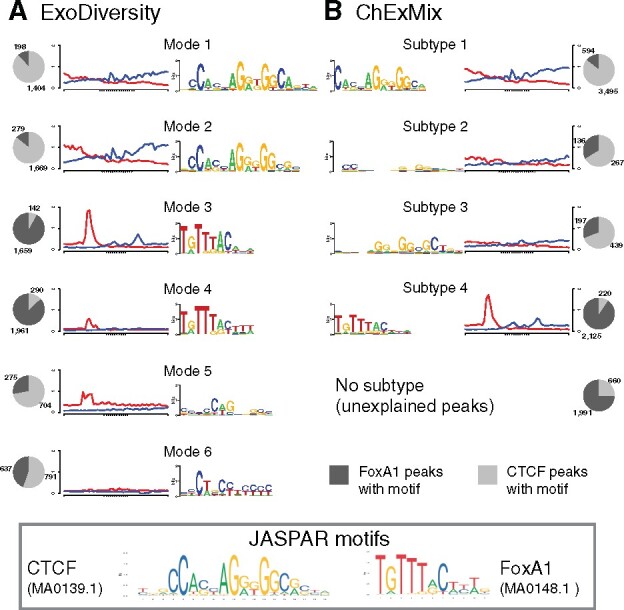
Motifs found in a dataset with 5000 FoxA1 and 5000 CTCF regions in the MCF7 cell-line. Note that unlike in ExoDiversity, the numbers in the pie charts for ChExMix do not add up to 10000, because it finds multiple subtypes in some regions and none in others

ExoDiversity should ideally be evaluated on a ChIP-exo dataset where the true footprints are well-established or on a simulated dataset where they could be artificially planted. However, the former does not currently exist and the latter will be constrained by assumptions of the planter, which may never match reality. We therefore followed the methodology of Yamada *et al.* (2019) where they mix two ChIP-exo datasets for TFs with well-established direct binding motifs: CTCF and FoxA1. Here, the peak regions were individually processed and 5000 such regions of each experiment were mixed. The goal is to see if CTCF and FoxA1 footprints can be resolved from the mixture of 10000 regions and the combined reads. ExoDiversity finds a total of six modes: the first two resemble the motif of CTCF (over 85% of the sites are in CTCF peaks), the next two are variants of the FoxA1 motif (almost 90% of the sites are in FoxA1 peaks), while the last two do not resemble any known motif, but explain the remaining 24% of the 10000 sequences (Fig. 2A). Of these, mode 5 is accompanied by a strong positive read signal five bases upstream, in contrast to mode 6, which has no enrichment of reads.

We note that modes 1 and 2 differ in the 3′ region of the motif: the existence of these nucleotide dependencies have been shown before (Eggeling *et al.*, 2014; Narlikar, 2013). Interestingly, the read footprints are different in intensity within both, the CTCF and the FoxA1 variants: modes 2 and 3 have stronger read signals than their variants, modes 1 and 4, respectively. The only other approach that detects subtypes of binding in ChIP-exo experiments without use of a motif-database, i.e. ChExMix, finds four subtypes: the largest is a clear match to CTCF, and like the first two modes of ExoDiversity has over 85% of the sites in CTCF peaks. The next two are weak motifs, which bear some resemblance to CTCF, but are less specific to CTCF peaks. The fourth subtype matches the FoxA1 motif and is enriched in the FoxA1 peaks, but explains only 42.5% of the total FoxA1 peaks, in contrast to over 70% of FoxA1 peaks getting explained by one of modes 3 or 4 of ExoDiversity. Overall, the motifs learned by ChExMix are weaker in terms of information content than those learned by ExoDiversity. We suspect this may be because ChExMix uses the motif discovery method MEME in each subtype to find motifs in the top 500 regions, but when extended to the full set of regions the motif becomes weaker. That said, the total number of peaks not explained by a CTCF or FoxA1 motif are only a little different between the two methods (2407 for ExoDiversity and 2651 for ChExMix).

### 4.2 ExoDiversity discovers 12 modes in Sox2 data

**Fig. 3.**
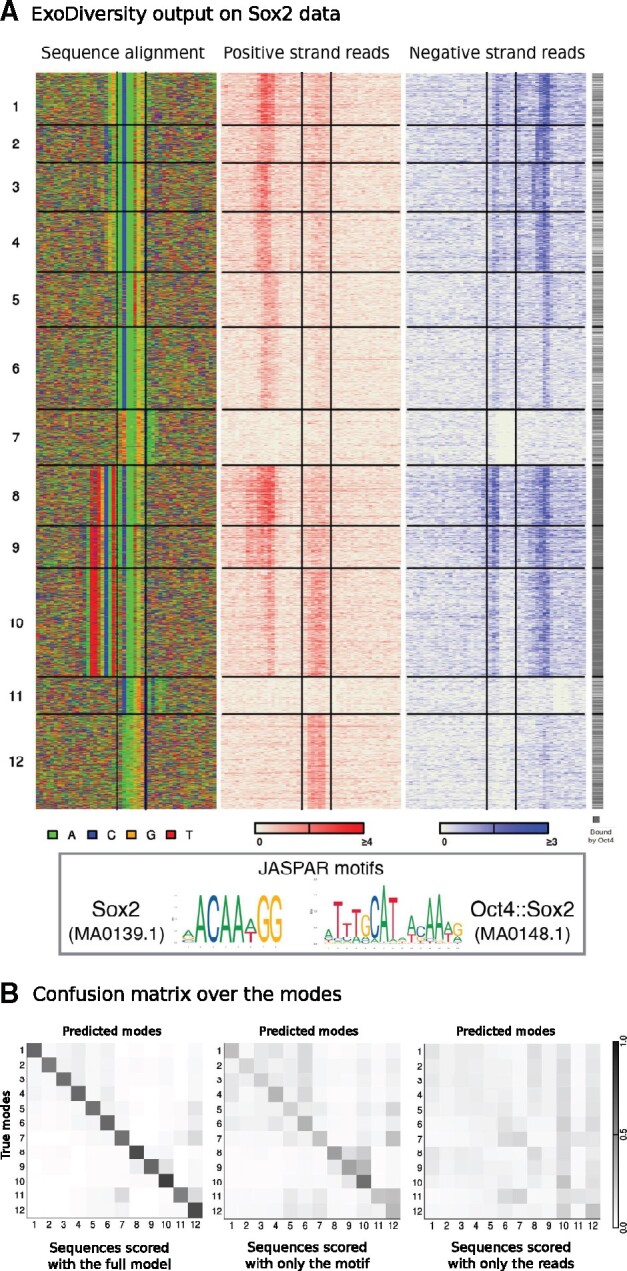
ExoDiversity finds 12 modes in the Sox2 dataset. (**A**) The DNA sequence and the two sets of reads in the 50 bp neighborhood are shown, with the central two lines in each plot marking the site corresponding to the Sox2 motif. The last column indicates which of the regions overlap with the peaks reported in the Oct4 ChIP-nexus experiment. (**B**) The three matrices show the probability distribution of a sequence over the 12 possible modes, averaged over sequences in each mode. The first matrix considers the full model, while other two use only one of the components: sequence or reads (Methods)

We next applied ExoDiversity to 7465 ChIP-nexus regions targeting Sox2 in murine embryonic stem-cells (ESCs) (Avsec *et al.*, 2021). Sox2 is essential for maintaining pluripotency in ESCs and recognizes two types of motifs shown in the box in Figure 3. ExoDiversity splits the data into a total of 12 modes, the first seven of which match the Sox2 motif. They differ in their 5′ flanking DNA as well as the read signal, which shifts by one or two positions on either strand. This suggests that the Sox2-DNA binding at the motif variants is different structurally. Further, the ChIP enrichment scores as reported by MACS at these modes are different (Supplementary Fig. S1), with the first mode having a significantly higher Sox2 occupancy (Wilcoxon *P*-value $<10^{-10}$). Modes 8–10 match the Oct4::Sox2 dimer motif; these modes are also significantly more occupied by Oct4 (grey plot in Fig. 3). They have differing Sox2 occupancies, with mode 8, like mode 1, having a significantly higher Sox2 occupancy (Wilcoxon *P*-value $<10^{-10}$, Supplementary Fig. S1). Curiously, although these motifs when compared to modes 1–7 have an additional Oct4 motif on the 5′ region, the positive reads do not appear to be shifted. This suggests that although Oct4 presumably does not bind to the modes 1–7, the exonuclease activity is nevertheless hindered similar to the locations where Oct4 does bind. Perhaps the position of the Sox2 protein is enough to obstruct the exonuclease from digesting that part of the DNA. Modes 11 and 12 are not obvious matches to any database motifs, however, the accompanying strong positive read signal for mode 12 suggests a potential experimental bias or artefact.

Visually, many modes appear similar. To quantify this similarity, we estimated posterior probabilities in three different ways. First, we calculated the probability of every datapoint belonging to each mode based on its corresponding reads and sequence (Equation 6, Supplementary Fig. S2). Unsurprisingly, the ‘true’ mode is always the most probable. We did a similar exercise using only the sequence information (***X*** and ϕ) and then using only the read information ($R^{+},\;R^{-},\;\phi^{+}$ and $\phi^{-}$). The mean probability of sequences of a particular mode predicted to belong to other modes, using each of these scoring techniques, is represented as three separate confusion matrices (Fig. 3B). Modes 1–7 and 8–10, which are variations of Sox2 and Oct4::Sox2 motifs, respectively, show only minor similarities when the full model is used. However, the similarity is more pronounced when only the motif information is used, as one would expect. The reads contribute little by themselves, but together with the motif information, the bold diagonal of the first matrix suggests that the modes are distinctly defined.

We also ran ExoDiversity on 19 051 ChIP-nexus regions targeting Oct4. It again finds several variants of Oct4, Oct4::Sox2 and Sox2 motifs, with different read footprints. Modes with the Oct4::Sox2 motif are most highly enriched with Oct4 occupancy. In addition, ExoDiversity finds motifs of Esrrb, Klf4 and CTCF, all of which are active in the ESCs (Supplementary Fig. S3). All these modes have relatively weak read signals, suggesting indirect binding.

### 4.3 ExoDiversity models can distinguish between sets bound by TFs with similar database motifs

We were struck by the large number of diverse modes captured by ExoDiversity in the Sox2 and Oct4 datasets. Although the two datasets overlap to a great extent, with the Oct4::Sox2 motif variant being enriched in the overlaps (Fig. 3), the individual modes are different in number and composition between the sets. While the modes are indeed distinct in probability space, we wanted to ensure they were not a result of over-fitting. One way to assess this is to see how general the model is in predicting unseen bound regions. The formulation of ExoDiversity enables us to find log likelihood ratios for a datapoint with respect to two models (Eq. 9).

**Fig. 4.**
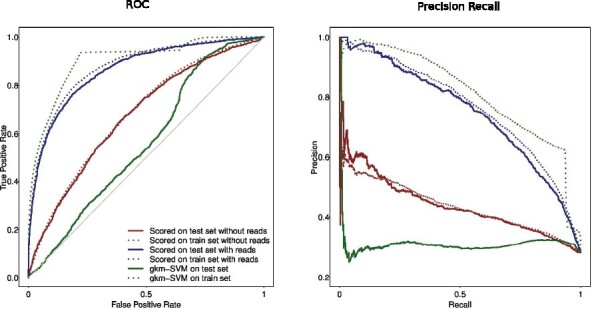
Performance of ExoDiversity-based classifier and gkm-SVM on distinguishing between Sox2-bound and Oct4-bound regions. The programs were trained on 80% of the dataset, with ExoDiversity being supplied the reads at those regions as well. The average area under the curves across all five folds for the different programs is shown in Supplementary Figure S4

We set up binary classification using Sox2 and Oct4 as our two classes of data. Standard five-fold cross-validation was applied, where ExoDiversity was trained on each fold of each TF separately using their respective summit regions and reads. The held out sets for both the TFs were scored using the best models learned by ExoDiversity for Sox2 and Oct4. We used two scoring systems: one which used the reads ($R^{+}$ and $R^{-}$) in addition to the DNA ***X***, and the other used only ***X***. Considering Sox2 as the positive and Oct4 as the negative class, we plot the receiver operating characteristic (ROC) curve and the precision-recall curve for one representative fold (Fig. 4). When the reads of the test set are used to score, as expected, the performance in distinguishing the sets is higher. But one may attribute that to the difference in the read counts, which would naturally be high for Sox2 at the Sox2 bound regions and vice versa. Indeed, when only the sequence component is used for scoring, the performance goes down. But it is still higher than the state-of-the-art SVM based classifier gkm-SVM, which goes beyond single PWMs, and using gapped k-mers distinguishes between functional regulatory elements to a remarkable degree (Ghandi *et al.*, 2014). This shows that although we are finally using only the sequence features for classification, the reads data used to train by ExoDiversity is critical in identifying those features. To test whether the BIC-selected model was not overfitting by identifying excessive modes, we also scored with the models learned with fewer modes. Those models also do better than gkm-SVM, but worse than the BIC-selected model (Supplementary Fig. S4). We also note that the performance of gkm-SVM on the training data is significantly higher than that on the test set; the difference is lower in the models learned by ExoDiversity (dotted curves in Fig. 4). This further confirms that the model is not over-fitting the training data.

### 4.4 ExoDiversity finds diverse binding modes in FoxA1 and CTCF datasets

We applied ExoDiversity to the complete FoxA1 data (40 013 sequences) in MCF7 cells, where it finds 13 modes (Supplementary Fig. S5), 11 of which are variants of the FoxA1 motif. The first two modes have an additional C three (mode 1) or two (mode 2) bases upstream of the canonical motif. Of these, the second mode has been shown to be enriched in regions bound by both FoxA1 and its paralog FoxA2 (Bochkis *et al.*, 2012). The position of the reads on the positive strand also moves correspondingly upstream when compared to mode 5, which is the best match to the canonical motif (box in Fig. 2). Similarly, modes 7 and 9 lack a strong A at the start and the corresponding cut goes a base deeper towards the motif. Strikingly, having *more* informative positions in the motif, although does result in small shifts in the read patterns, does not necessarily correlate with more reads. This is counter-intuitive since one expects to see higher occupancy of the TF at motifs with more information content. We surmise that these regions may also be bound by other proteins (e.g. FoxA2 in mode 2), and due to the resulting competition between FoxA1 and other TFs for the site, fewer instances of the POI-DNA complex are pulled down, causing fewer reads.

**Fig. 5.**
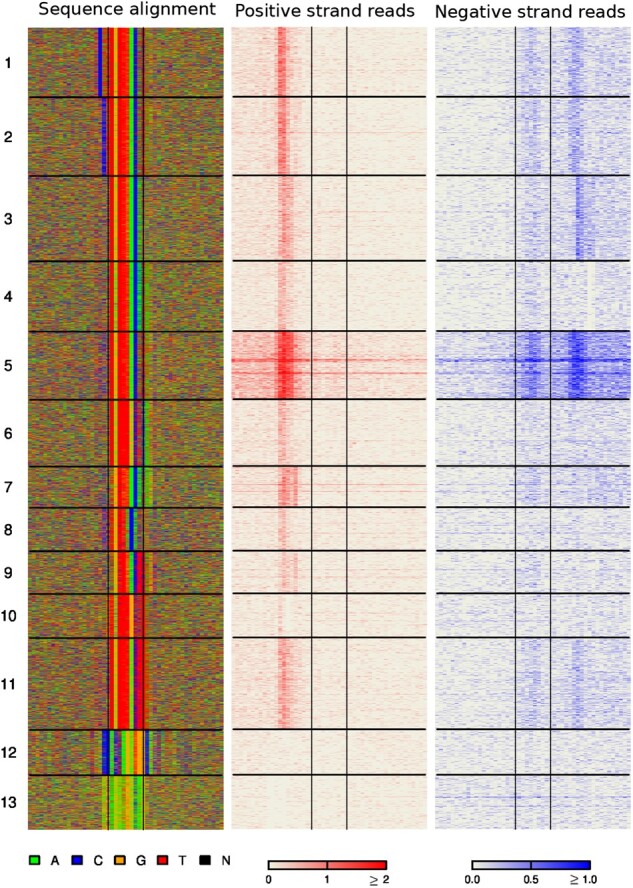
ExoDiversity finds 13 modes in the FoxA1 dataset. The DNA sequence and the two sets of reads in the 50 bp neighborhood are shown, with the central two lines in each plot marking the site corresponding to the FoxA1 motif

The last two modes are a low complexity region (mode 13) and the CTCF motif (mode 12), both of which are associated with very few reads. CTCF is known to be interact with FoxA1 and indirect binding between them has been suggested before (Yamada *et al.*, 2019).

**Fig. 6.**
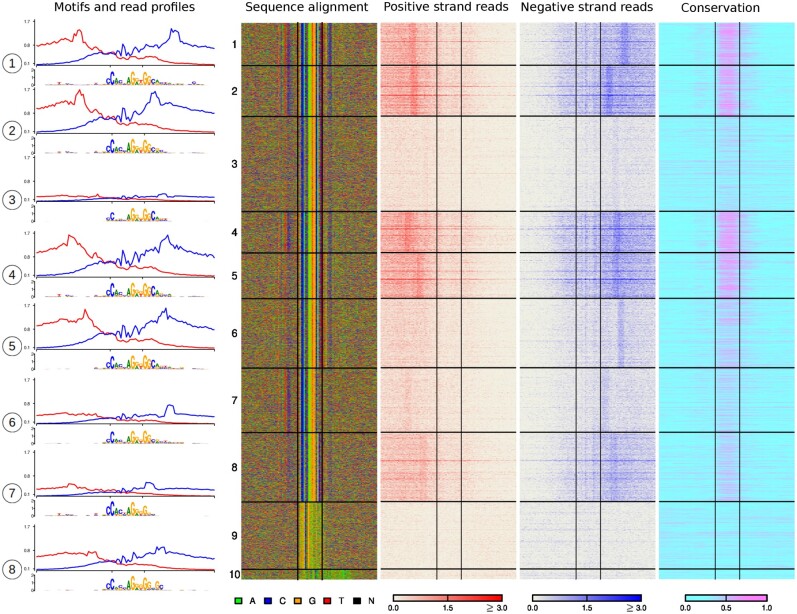
ExoDiversity finds 10 modes in the CTCF dataset. The read profiles from both the positive strand (red) and negative strand (blue) over a 100 bp window about the motifs along with motifs learned by ExoDiversity on the eight modes that resemble the canonical CTCF motif are shown in the left. The DNA sequence, the two sets of reads and the corresponding sequence conservation in terms of phastCons scores in the 100 bp neighborhood are shown. The X-axes of the read profiles are aligned corresponding to the 19 bp JASPAR CTCF motif (Fig. 2), which is also shown with a box in the middle of the four heat-map images and two ticks in the read profile. Note that the read profile is over a 100 bp region and the motif shown below is over a 40 bp region

CTCF is a protein with 11 zinc fingers and while it binds to a core motif shown in Figure 2, it employs its zinc fingers to bind DNA in multiple ways. Two specific motifs on each side of the core have been shown to provide additional stability to the CTCF-DNA complex (Nakahashi *et al.*, 2013). In order to capture these, we applied ExoDiversity to the CTCF set (55 885 regions) in the same cells with an initial width of 40. It finds eight modes that resemble the CTCF core motif, with differing strengths of the additional flanking motifs (Fig. 6). Indeed, modes that contain at least one of the additional motifs, are marked by high intensity positive/negative reads at *both* ends, although the position of the reads changes depending on the strength of the additional motifs. For example, modes 1 and 2 both have the upstream motif, but mode 2 has a much weaker downstream motif: the negative strand reads are correspondingly shifted towards the core in this mode. Similarly, the core motif misses a few downstream bases in mode 7, but is associated with a band of reads on both strands (albeit weak); in contrast, although the core motif is fairly well conserved in mode 3, the bands of reads are missing. The only other difference between the two modes is the presence of the upstream motif in mode 7. Furthermore, sequence conservation varies across the modes (modes 1, 2, 4, 5 are significantly more conserved, while modes 3, 6–8 are less; Wilcoxon *P*-value $<10^{-10}$), which generally correlates with the number of reads associated with the modes. It does not necessarily correlate with the information content of the core motif.

### 4.5 Twist

**Fig. 7.**
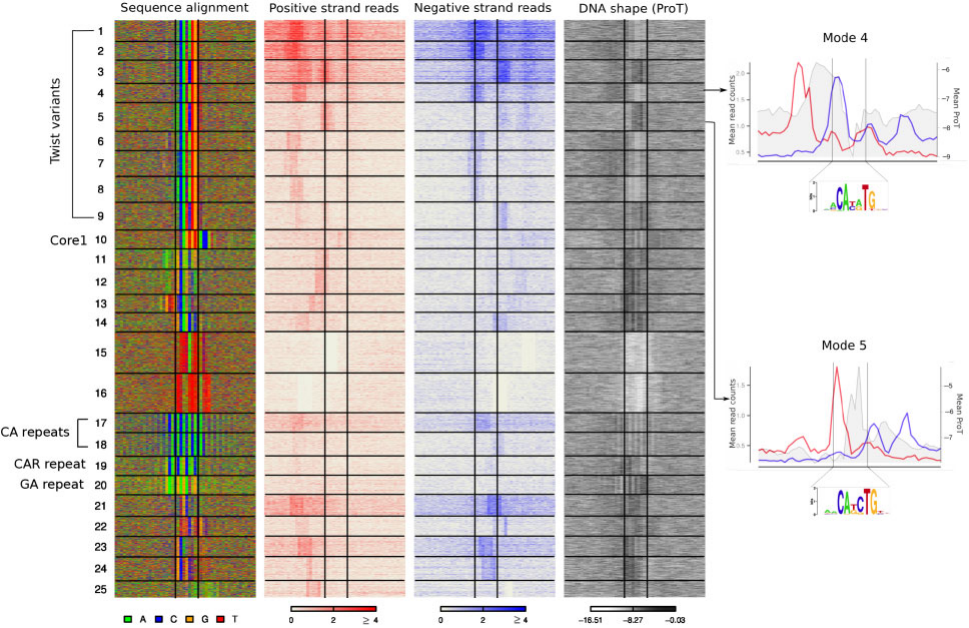
ExoDiversity finds 25 modes in the Twist dataset. Modes 1–9 correspond to Twist motifs; Mode 10 matches a *Drosophila* core promoter motif and Modes 17–20 are di/tri-nucleotide repeat patterns. The DNA sequence, the two sets of reads and the DNA shape for propeller twist in the 50 bp neighborhood are shown, with the central two lines in each plot marking the site corresponding to the Twist motif. Two representative modes for the two Twist variants are shown on the right. The mean positive and negative reads are shown in red and blue plots, respectively (left *Y*-axis). The mean propeller twist values are shown in grey (right *Y*-axis)

We next applied ExoDiversity to 29 353 ChIP-nexus regions from the E-box binding TF Twist in early Drosophila embryos (He *et al.*, 2015). It finds a total of 25 modes, of which nine match the E-box (Fig. 7). The authors had shown that while overall, three peaks of reads are visible around the bound E-box, the individual peaks are specific to certain E-box core motifs: the first peak is enriched about 11 bp upstream of CA**YA**TG and the second is enriched 2 bp inside CA**KC**TG. That analysis was done using the top 200 most enriched regions for each E-box variant. ExoDiversity also identifies similar correlations from the complete dataset, without any prior information about the E-box. It finds additional variations of small shifts in the first peak which co-occur with small differences within and around the E-box.

Genomic regions flanking E-box binding sites have been shown to possess specific local DNA shapes that correlate with binding (Gordan *et al.*, 2013). The propeller twist of the DNA, which denotes the rotation of one base with respect to its base-pair has been shown to be important for other proteins recognizing E-boxes (He *et al.*, 2015). We therefore plotted computationally predicted propeller twist (proT) (Chiu *et al.*, 2015) at the DNA for each mode. Motifs at modes 1, 2, 4, 6–8, which correspond to the CA**YA**TG E-box have a lower propeller twist than the motifs at modes 3, 5 and 9, which correspond to the CA**KC**TG E-box. Since these DNA shape measures are based on the sequence, it is not surprising that the two variants have strikingly different shape profiles. However, the upstream flanks, which do not have a strong motif signal, possess a higher propeller twist at the CA**YA**TG modes than the CA**KC**TG modes. Curiously, the signal of reads on the positive strand aligns 7–10 bp upstream of the positions with high propeller twist across the E-box modes. Similarly the signal on the negative strand aligns 7–10 bp downstream of the high propeller twist regions (Fig. 7). A high propeller twist indicates more DNA rigidity, which might explain the differing positions of the read signal and their intensities at the nine modes.

The Twist modes explain less than half of the total reported regions. ExoDiversity finds 16 modes that do not have E-box motifs, but many of which are well-studied fly motifs. For instance, mode 10 matches the Drosophila core promoter motif 1 (Ohler *et al.*, 2002). Indeed, the median distance of the sequences in that mode from the closest transcription start site is 25 bp (Wilcoxon *P*-value $<10^{-10}$ when compared to the rest of the set). On the other hand, modes 17–20 contain specific di- and tri-nucleotide repeat patterns, which are characteristic of fly enhancers (Brittain *et al.*, 2014; Yanez-Cuna *et al.*, 2014). Considering that these non-Twist motifs have far fewer reads overall, they are likely indirect binding events involving Twist.

## 5 Discussion

ExoDiversity resolves protein-DNA binding footprints by learning a joint distribution over DNA regions and corresponding read counts arising from exonuclease-based ChIP experiments. On a range of ChIP-nexus and ChIP-exo datasets, we showed that it detects variations within the DNA motif, which correlate with read signals in the immediate neighborhood. We further showed that some of the detected modes have distinct characteristics such has higher/lower sequence conservation, specific DNA shapes and proximity to transcription start sites. We note that while we cannot definitively establish whether the variations in all the detected modes are biologically relevant, these modes together are needed to describe the whole dataset. The ROC curves demonstrate that the models are not a result of overfitting. Indeed, ExoDiversity is unique in its goal of finding an explanation for each reported region. It therefore identifies potential cofactors like CTCF and Esrrb in Oct4, which have relatively poor read signals and make up a small fraction of the total data.

Currently we use non-informative priors for all parameters in the model. However, the Bayesian formulation allows one to incorporate prior knowledge, if available. For example, if the structural family of the profiled TF is known, one could use motif priors corresponding to its family-motifs. This might shed more light on potential indirect binding modes. We also note that motifs that are not obvious TF binding sites should be explored for possible experiment biases, especially when they are accompanied by strong read signals.

ChExMix is another method that attempts to find subtypes based on motifs and reads. However, it is different from ExoDiversity in its problem formulation. First, it is also a peak or event finder, and therefore at times detects binding events well over 500 bp away from the input peak region. Second, it treats each subtype independently, in a way, so allows for multiple subtypes to occur in a region as well as some regions to not have any binding event. Finally, motif discovery is conducted only in the top few regions of each sub-type. Indeed, most downstream motif-based analyses focus on the top *x* regions, either because of the time taken to analyze the full set, or because those regions have a higher likelihood of containing the expected motif. ChExMix uses these strong motifs for subsequent scanning or as priors, but this causes the final motifs, those made by all the final predicted binding events to be weaker (Supplementary Figs S1, S3, S5 and S6), giving an average description of the dataset. In contrast, because ExoDiversity maximizes a joint distribution, its modes are not necessarily guided only by strong reads or enriched regions. This, we believe is critical, since regions with lower read counts are not necessarily uninformative. At times the motif slightly differs from the canonical one, but affects exonuclease activity, or it may be a motif of a different protein, but captured due to its association with the POI. We also note that unlike ChExMix, we focus on a narrow window of reads (5 bp here) in the immediate neighborhood of the motif. As a result, ExoDiversity’s modes are not only characterized by exonuclease cuts differing at the level of a few nucleotides, but also have stronger corresponding motifs, when compared to ChExMix. Indeed, when we run ExoDiversity with larger width values (10, 50, 100), we notice that while the results for 10 are not too different from five, the motifs get progressively weaker for larger read widths (Supplementary Figs S7, S8). This is because the read distribution starts dominating over the motif information, effectively making a larger contribution to the joint distribution of eq. (1). ExoDiversity gives the user the option of supplying a read width of their choice; however, learning the read width in a manner similar to the motif width is a promising extension.

Avsec *et al.* (2021) use BPNet, a deep learning based approach, which trains on ChIP-nexus profiles and predicts profiles for unseen DNA regions to a remarkable degree. Their primary goal is to decipher the syntax or the architecture of TF binding sites within regulatory regions, and not specifically to resolve footprints. We note that ExoDiversity is currently limited to identifying one mode in each region. Changing the model to a module finder, based on combinations of binding modes (Biswas and Narlikar, 2020), will be useful. Further, ExoDiversity approximates the read distributions as a product of independent Bernoulli distributions, conditional on the mode. The assumption here is that the cleaving activity, i.e. location where the cut happens, depends only on the type of complex forming at the DNA. This effectively results in an extension of the PWM model, which also assumes independence between positions of the binding site. However, other approximations as well as different distributions which overcome this assumption are worth exploring.

We note that because ExoDiversity relies heavily on read information at the nucleotide level, it is not applicable to the more popular ChIP-seq protocol which reports wider read distributions. However, several modifications have been suggested recently to the original ChIP-exo protocol, which may result in a broader adoption of the technique, substituting ChIP-seq (Rossi *et al.*, 2018). We therefore envision ExoDiversity being used more widely (Supplementary Figs S9–S12 show results on four other TFs–Nanog, Klf4, GR and ER*α*–not discussed here). That said, the formulation behind ExoDiversity can be extended to other sources of information, which are of high resolution and are indicative of DNA motifs in the neighborhood, such as transcription start site data or sequence conservation information.

## Supplementary Material

btab274_Supplementary_Data
