# Supplementary material for: Resolving diverse protein–DNA footprints from exonuclease-based ChIP experiments
Source: Bioinformatics. 2021 Jul 12;37(Suppl 1):i367–75. doi: 10.1093/bioinformatics/btab274 (PMC8275329; doi:10.1093/bioinformatics/btab274)
Supplement: btab274_Supplementary_Data [file btab274_supplementary_data.pdf]

1 Supplementary Methods

1.1 Model Description

Recall that from the experiment, we have the following:

- n reported DNA sequences $\boldsymbol{X} = \boldsymbol{X_1}, \boldsymbol{X_2}, \ldots, \boldsymbol{X_n}$. Here $X_{i,j}$ is one of {A,C,G,T,N}; $1 \leq j \leq L_i$ where L_i is the length of $\boldsymbol{X_i}$.
- n vectors $\boldsymbol{R}^+ = \boldsymbol{R_1^+}, \boldsymbol{R_2^+}, \ldots, \boldsymbol{R_n^+}$, which store read counts corresponding to $\boldsymbol{X}$ on the positive strand. $R^+_{i,j}$ denotes the number of reads whose $5'$ end maps to the genomic location corresponding to $X_{i,j}$ on the positive strand. These counts are discretized to binary values based on the median read count or a user-defined threshold.
- n vectors $\boldsymbol{R}^-$ similar to above but which store read counts mapping to the negative strand.

The goal is to partition the dataset into m binding modes i.e., learn parameters $\boldsymbol{\theta}_m$ of a model $\boldsymbol{M}_m$, which has six components $\{\boldsymbol{Z}, \boldsymbol{I}, \boldsymbol{w}, \boldsymbol{\phi}, \boldsymbol{\tau}, \boldsymbol{\gamma}\}$:

- Z_i: position of the motif in $\boldsymbol{X_i}$
- I_i: binding mode of $\boldsymbol{X_i}$; $1 \leq I_i \leq m$
- w_k: width of the PWM motif in mode k; $1 \leq k \leq m$
- w_k^+: width of the read window defining the positive strand footprint in mode k; here it is set to 5, but can be changed by the user
- w_k^-: as above, but for the negative strand
- $\boldsymbol{\phi_k}$: PWM values for motif of mode k. $\phi_{k,a}(b)$ is the probability of finding nucleotide b at position a in PWM of mode k; $1 \leq a \leq w_k$
- $\boldsymbol{\phi_k^+}$: Bernoulli probabilities of finding a read on the positive strand for mode k. $\phi^+_{k,a}(1)$ is the probability of having a positive strand read at position a within the read window of mode k, while $\phi^+_{k,a}(0) = 1 - \phi^+_{k,a}(1)$ is the probability of finding no read there; $1 \leq a \leq w_k^+$
- $\boldsymbol{\phi_k^-}$: Bernoulli probabilities for negative strand reads in mode k similar to above
- $\boldsymbol{\phi_0}$: parameters of the background distribution over the DNA sequences (2nd order Markov model learned directly from $\boldsymbol{X}$)
- $\boldsymbol{\phi_0^+}$ and $\boldsymbol{\phi_0^-}$: parameters of the background strand read distribution (0th order Markov model learned directly from $\boldsymbol{R}^+$ and $\boldsymbol{R}^-$, respectively)

- τ_k^+: distance of the start of positive strand read window from the start of motif in mode k (positive read window offset)

- τ_k^-: distance of the start of the motif from the start of the negative strand read window in mode k (negative read window offset)

- $\boldsymbol{\gamma}$: Categorical distribution over m modes. γ_k is the probability of a sequence containing the binding mode k.

1.2 Model Learning

The goal is to learn the parameters of the model $\boldsymbol{M}_m$ to maximize the posterior distribution:

$$\arg\max_{\boldsymbol{\theta}_m} P(\boldsymbol{\theta}_m \mid \boldsymbol{X}, \boldsymbol{R}^+, \boldsymbol{R}^-, \boldsymbol{M}_m) \tag{1}$$

$\boldsymbol{\theta}_m$ is a high dimensional vector. We therefore use collapsed Gibbs sampling targeting a marginalized posterior, as has been done previously with motif discovery [Liu, 1994, Mitra et al., 2018]. Here, we integrate out (marginalize over) the $\boldsymbol{\phi_k}, \boldsymbol{\phi_k^+}, \boldsymbol{\phi_k^-}$ and $\boldsymbol{\gamma}$, and iteratively sample the other parameters: locations of motifs (Z_i) & mode of binding (I_i) for each datapoint i and read window offsets (τ_k^+, τ_k^-) and width of motifs (w_k), for each binding mode k. Background probabilities $\boldsymbol{\phi_0}, \boldsymbol{\phi_0^+}, \boldsymbol{\phi_0^-}$, which describe the regions without motif/read windows are pre-calculated from $\boldsymbol{X}, \boldsymbol{R}^+, \boldsymbol{R}^-$ as mentioned in Methods (main paper). We assume conjugate priors for ease of integration: a Dirichlet prior over each position of $\boldsymbol{\phi_k}$ with equal hyperparameters α_{motif}, a Beta prior over each position of $\boldsymbol{\phi_k^+}$ and $\boldsymbol{\phi_k^-}$ with equal hyperparameters α_{read}, and a Dirichlet prior over $\boldsymbol{\gamma}$ with equal hyperparameters α_{mode}. These hyperparameters can be changed by the user, but by default we use the non-informative value of 1 for α_{read} and α_{mode}, and a sparsity promoting value of 0.5 for α_{motif}.

1.3 Sampling expressions

Using the notation $\boldsymbol{V}_{[-i]}$ to denote the vector $\boldsymbol{V}$ without the ith component, we get the following individual sampling expressions.

$$\begin{aligned} &P(Z_i = j \mid \boldsymbol{X}, \boldsymbol{R}^+, \boldsymbol{R}^-, \boldsymbol{Z}_{[-i]}, \boldsymbol{I}, \boldsymbol{w}, \boldsymbol{\phi_0}, \boldsymbol{\phi_0^+}, \boldsymbol{\phi_0^-}, \boldsymbol{\tau}^+, \boldsymbol{\tau}^-) \\ &\propto \frac{\prod_{a=1}^{w_{I_i}} \widehat{\phi}_{I_i,a}(X_{i,j+a-1})}{P(X_{i,j}, \ldots, X_{i,j+w_{I_i}-1} \mid \boldsymbol{\phi_0})} \times \prod_{s\in\{+,-\}} \frac{\prod_{a=1}^{w^s_{I_i}} \widehat{\phi}^s_{I_i,a}(R^s_{i,j+\tau^s_{I_i}+a-1})}{P(R^s_{i,j+\tau^s_{I_i}}, \ldots, R^s_{i,j+\tau^s_{I_i}+w^s_{I_i}-1} \mid \boldsymbol{\phi}^s_{\boldsymbol{0}})} \end{aligned} \tag{2}$$

where $\widehat{\phi}_{I_i,a}$ and $\widehat{\phi}^s_{I_i,a}$ are the posterior means of $\phi_{I_i,a}$ and $\phi^s_{I_i,a}$ conditional on the current values of all the parameters except the ith datapoint [Liu et al., 1995]:

$$\widehat{\phi}_{I_i,a}(B) = \frac{\alpha_{\text{motif}} + \sum\limits_{\substack{p=1 \\ p \neq i}}^{n} \delta[X_{p,Z_p+a} = B, I_p = I_i]}{4\alpha_{\text{motif}} + \sum\limits_{\substack{p=1 \\ p \neq i}}^{n} \delta[I_p = I_i]} \tag{3}$$

$$\widehat{\phi}^{s}_{I_i,a}(A) = \frac{\alpha_{\text{read}} + \sum\limits_{\substack{p=1 \\ p \neq i}}^{n} \delta[R^{s}_{p,Z_p+\tau^{s}_{I_i}+a} = A, I_p = I_i]}{2\alpha_{\text{read}} + \sum\limits_{\substack{p=1 \\ p \neq i}}^{n} \delta[I_p = I_i]} \tag{4}$$

$\delta[\textit{condition}] = 1$ when *condition* is TRUE and 0 otherwise.

The binding mode k of a datapoint is sampled in a similar manner.

$$\begin{aligned} &P(I_i = k \mid \boldsymbol{X}, \boldsymbol{R}^{+}, \boldsymbol{R}^{-}, \boldsymbol{Z}, \boldsymbol{I}_{[-i]}, \boldsymbol{w}, \boldsymbol{\phi_0}, \boldsymbol{\phi^{+}_0}, \boldsymbol{\phi^{-}_0}, \boldsymbol{\tau}^{+}, \boldsymbol{\tau}^{-}) \\ &\propto \widehat{\gamma}_k \times \frac{\prod\limits_{a=1}^{w_k} \widehat{\phi}_{k,a}(X_{i,Z_i+a-1})}{P(X_{i,Z_i}, \ldots, X_{i,Z_i+w_k-1} \mid \boldsymbol{\phi_0})} \times \frac{\prod\limits_{a=1}^{w^{s}_k} \widehat{\phi}^{s}_{k,a}(R_{i,Z_i+\tau^{s}_k+a-1})}{P(R^{s}_{i,Z_i+\tau^{s}_k}, \ldots, R^{s}_{i,Z_i+\tau^{s}_k+w^{s}_k-1} \mid \boldsymbol{\phi^{s}_0})} \end{aligned} \tag{5}$$

where $\widehat{\boldsymbol{\gamma}}$ is the posterior mean of $\boldsymbol{\gamma}$ conditional on the current values of $\boldsymbol{I}$ except for the ith datapoint [Liu et al., 1995]:

$$\widehat{\gamma}_k = \frac{\alpha_{\text{mode}} + \sum\limits_{\substack{p=1 \\ p \neq i}}^{n} \delta[I_p = k]}{m\alpha_{\text{mode}} + n - 1} \tag{6}$$

τ^{+}_k and τ^{-}_k, which determine the position of the read windows with respect to the start of the DNA motif for mode k are sampled from all possible positions as long as the positive read window does not go beyond the motif's $3'$ end and similarly the negative read window does not precede the motif[1]. In that case the conditional probability of τ^{+}_k being l, where l is one of these positions can be computed as:

$$P(\tau^{+}_k = l \mid \boldsymbol{Z}, \boldsymbol{I}, \boldsymbol{X}, \boldsymbol{R}^{+}, \boldsymbol{R}^{-}) \propto \prod_{\substack{i=1 \\ I_i = k}}^{n} \prod_{a=1}^{w^{+}_{I_i}} \widehat{\phi}^{+}_{k,a}(R^{+}_{i,Z_i-l+a}) \tag{7}$$

where $\widehat{\phi}^{+}_{k,a}$ is similar to eq. (1.3) but over all the sequences.

$$\widehat{\phi}^{+}_{k,a}(A) = \frac{\alpha_{\text{read}} + \sum\limits_{p=1}^{n} \delta[R^{+}_{p,Z_p+\tau^{+}_k+a} = A, I_p = k]}{2\alpha_{\text{read}} + \sum\limits_{p=1}^{n} \delta[I_p = k]} \tag{8}$$

τ^{-}_k is sampled in a similar manner. The width w_k of motif k is sampled as done earlier [Mitra et al., 2018]: instead of sampling from a large set of possible values in one iteration, the width is allowed to increase

[1] The implementation on github includes a flag that allows for the positive (negative) read window to go beyond (precede) the $3'$ ($5'$) end, constraining only the start to lie before (after) the $3'$ ($5'$) end of the motif. All results in the paper are without this flag.

or decrease by one on each side of the motif, or stay the same. We notice the width does not change very often, so to speed up the program, it is sampled after the other variables are sampled 10 times. The initial width is set to 12 as default, but can be changed by the user. Each sampling run is executed from a default of 10 random initializations, but this number can also be changed by the user.

References

J. Liu. The collapsed gibbs sampler with applications to a gene regulation problem. *J. Am. Stat. Assoc.*, 89:958–966, 1994.

Jun S. Liu, Andrew F. Neuwald, and Charles E. Lawrence. Bayesian models for multiple local sequence alignment and gibbs sampling strategies. *J Am Stat Assoc*, 90(432):1156–1170, 1995. doi: 10.1080/01621459.1995.10476622.

Sneha Mitra, Anushua Biswas, and Leelavati Narlikar. Diversity in binding, regulation, and evolution revealed from high-throughput chip. *PLoS Comput Biol*, 14(4):e1006090, 2018.

2 Supplementary Figures

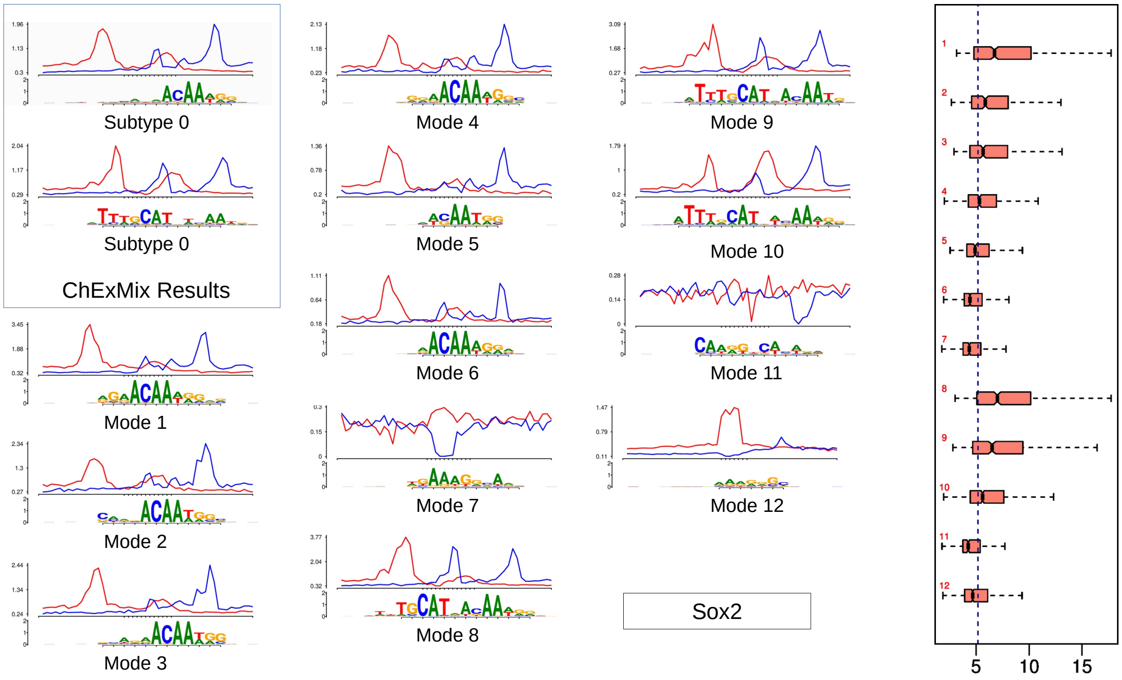

Fig. S1. The output on the Sox2 dataset is shown. ChExMix (top left) finds two motifs explaining 75% of the data, resembling Sox2 and Oct4::Sox2, respectively. ExoDiversity finds many more modes, which have differing enrichment scores as determined by MACS2 (boxplot on the right). The average read plots are computed over a 50bp neighborhood around the motif: the ticks correspond to the motif positions in that mode (ExoDiversity) or subtype (ChExMix) shown below the plot in each case.

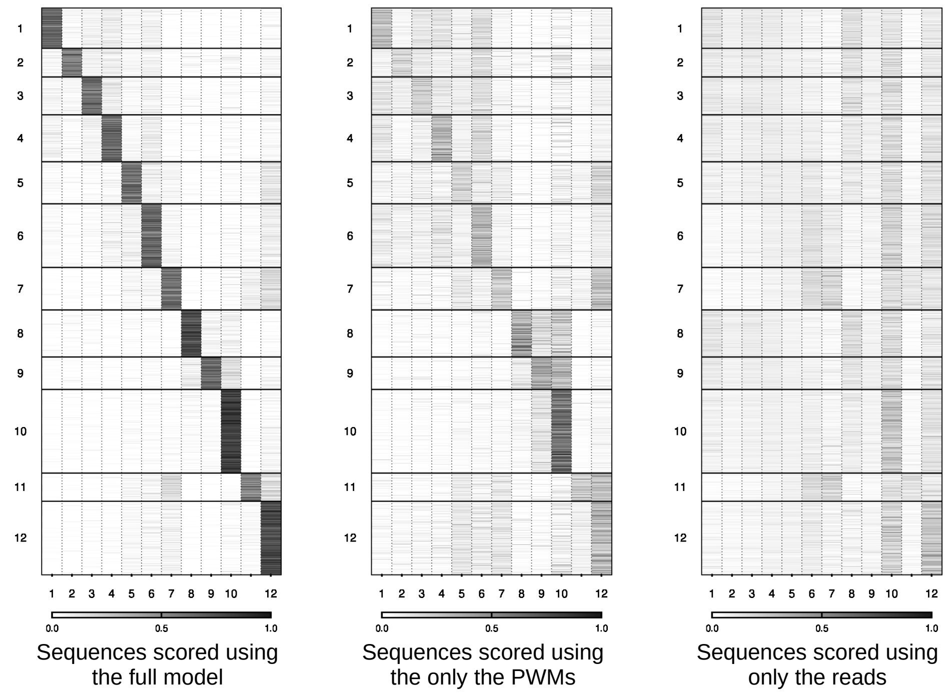

Fig. S2. Sequences in the Sox2 dataset are scored in three different ways: with the full model, with only the motif information, and with only the read information. Each row denotes a sequence the dataset ordered according to those in Fig. 3A. Each column k denotes the probability of that sequence being predicted to be in mode k (eq. 6 in the main text). The probabilities are distinctly higher (close 1) for the modes in the original model, showing the modes are indeed different. Using only the motif model shows that the first six modes have similarities, so do modes 8–10.

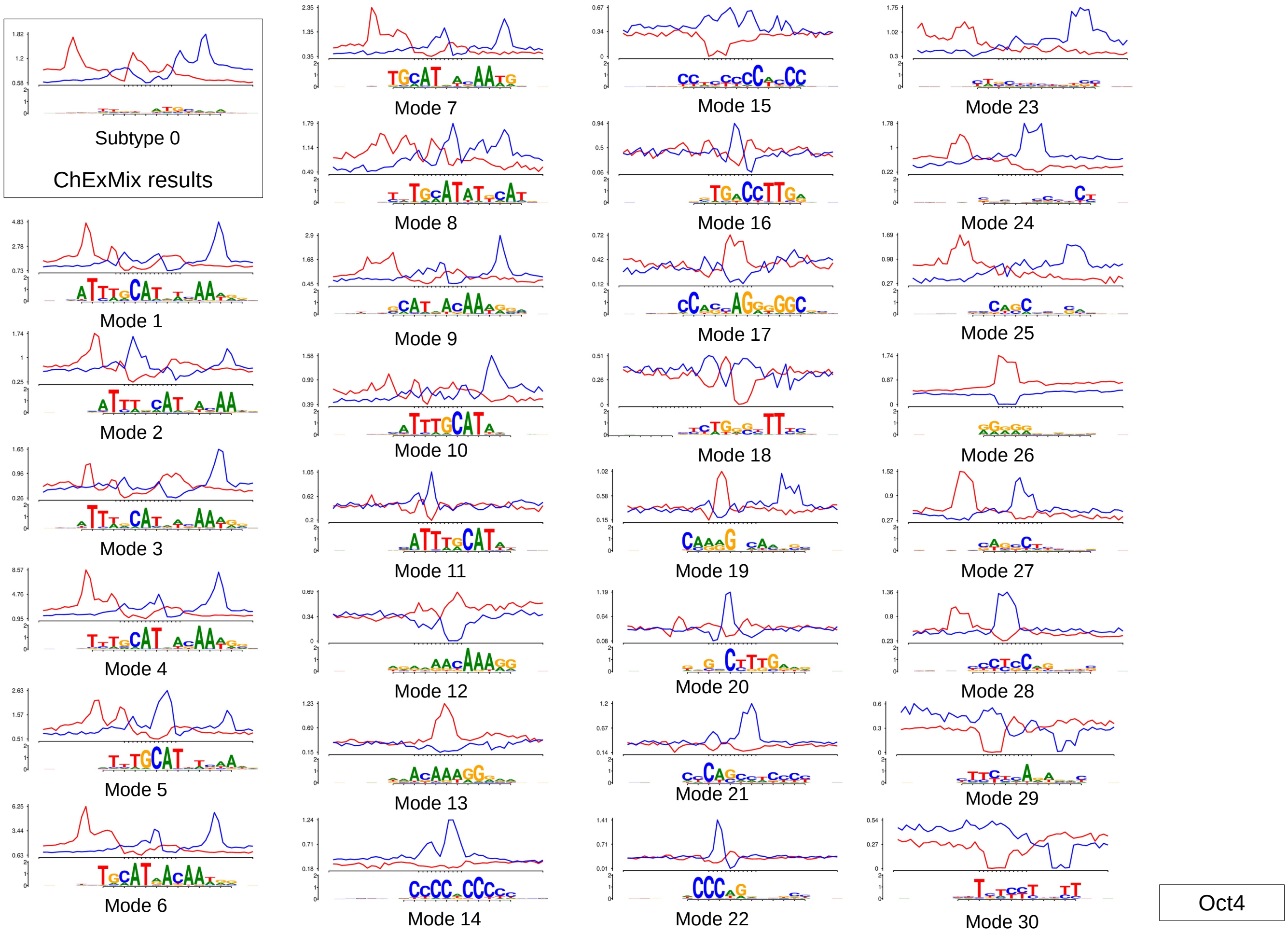

Fig. S3. The output on the Oct4 dataset is shown. ChExMix (top left) finds a single motif explaining 76% of the data, but resembles a very weak Oct4::Sox2 motif and low intensity read distributions. ExoDiversity finds many more modes, of which Oct4::Sox2 motifs are stronger and reads vary in intensity (modes 1–8), Oct4::Oct4 motif (mode 9), Oct4 motif (modes 10, 11) with different read positions, Sox2 motifs (modes 12, 13, 19, 20), Klf4 (modes 14, 15), Esrrb (mode 16), CTCF (mode 17) as well as other low complexity regions, some of which have high reads (Discussion). The average read plots are computed over a 50bp neighborhood around the motif: the ticks correspond to the motif positions in that mode (ExoDiversity) or subtype (ChExMix) shown below the plot in each case.

ROC

Average auROC	Score with reads			Score without reads			gkm-SVM
	BIC model	1 mode	3 modes	BIC model	1 mode	3 modes	
On test set	0.87	0.82	0.81	0.67	0.62	0.64	0.56
On train set	0.88	0.83	0.82	0.68	0.62	0.65	0.90

Precision Recall

Average auPRC	Score with reads			Score without reads			gkm-SVM
	BIC model	1 mode	3 modes	BIC model	1 mode	3 modes	
On test set	0.76	0.67	0.67	0.43	0.39	0.41	0.31
On train set	0.78	0.67	0.68	0.44	0.39	0.41	0.82

Fig. S4. The average areas under ROC and PR curves for classifying Sox2 versus Oct4, across five folds are shown. The BIC-based model performs the best with or without reads on the test data. On the training set gkm-SVM performs the best.

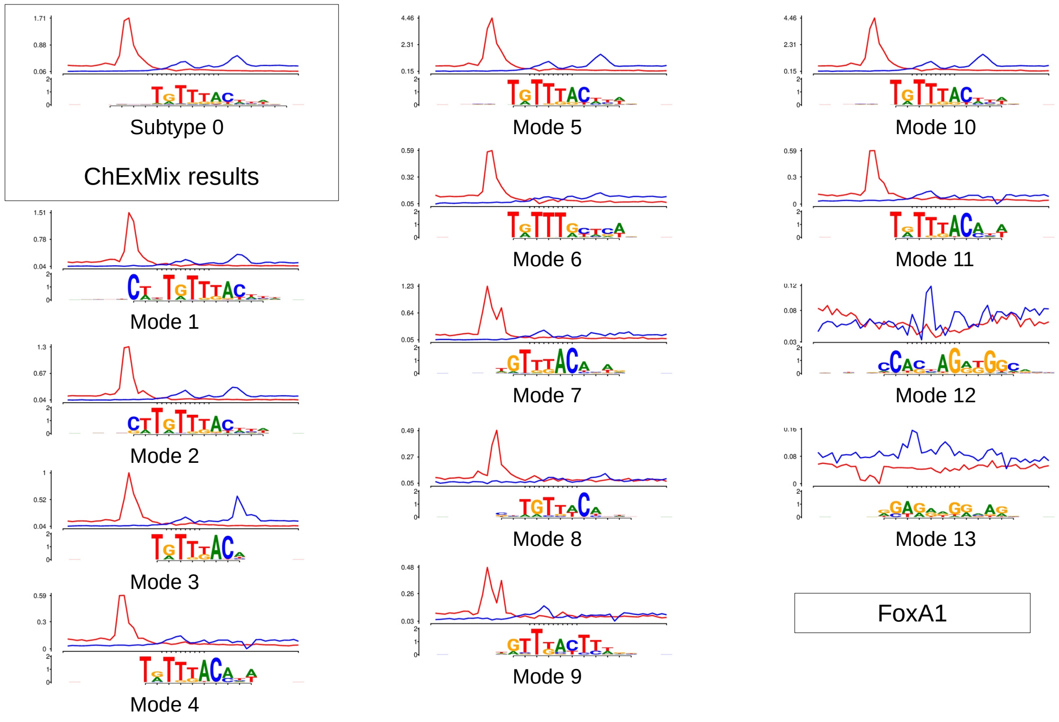

Fig. S5. The output on the FoxA1 dataset is shown. ChExMix (top left) finds a single FoxA1 motif and an additional subtype characterized only by the reads. ExoDiversity finds many more modes, of which mode 5 is the closest match to the canonical FoxA1, and also has much higher read counts. The average read plots are computed over a 50bp neighborhood around the motif: the ticks correspond to the motif positions in that mode (ExoDiversity) or subtype (ChExMix) shown below the plot in each case.

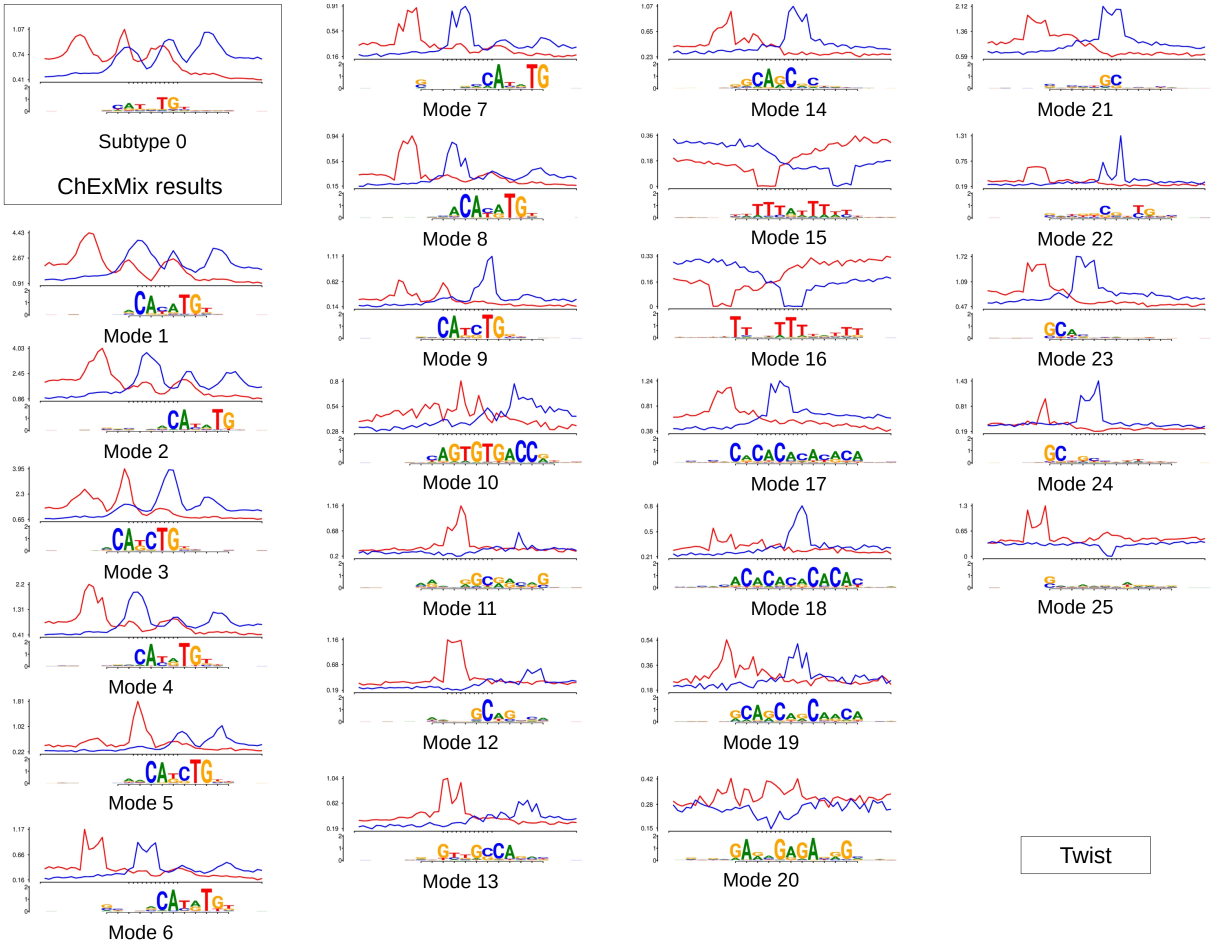

Fig. S6. The output on the Twist dataset is shown. ChExMix (top left) finds a single weak E-box motif. ExoDiversity finds nine E-box variants, with differing cores and read distributions. It additionally finds well-established fly motifs (main text) accompanied by specific read distributions. The average read plots are computed over a 50bp neighborhood around the motif: the ticks correspond to the motif positions in that mode (ExoDiversity) or subtype (ChExMix) shown below the plot in each case.

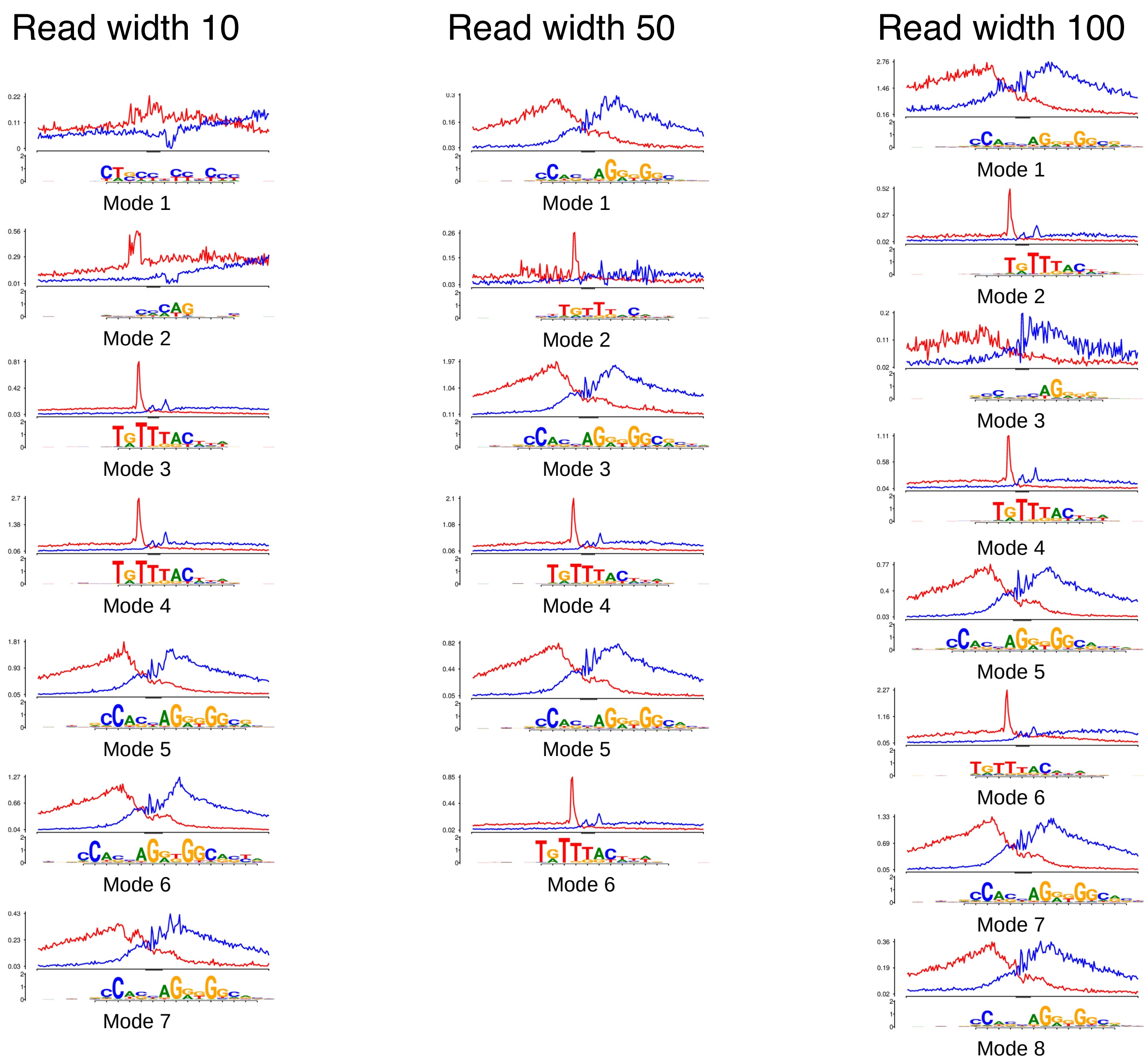

Fig. S7. ExoDiversity was run with three different values of read widths on the mixed dataset. The result of using $w_k^{+/-} = 10$ is not very different from using a value of 5 (Fig. 2 in main text). However, for values of 50 and 100, the motifs become weaker. The average read plots are computed over a 200bp neighborhood around the motif: the ticks correspond to the motif positions in that mode shown below the plot in each case.

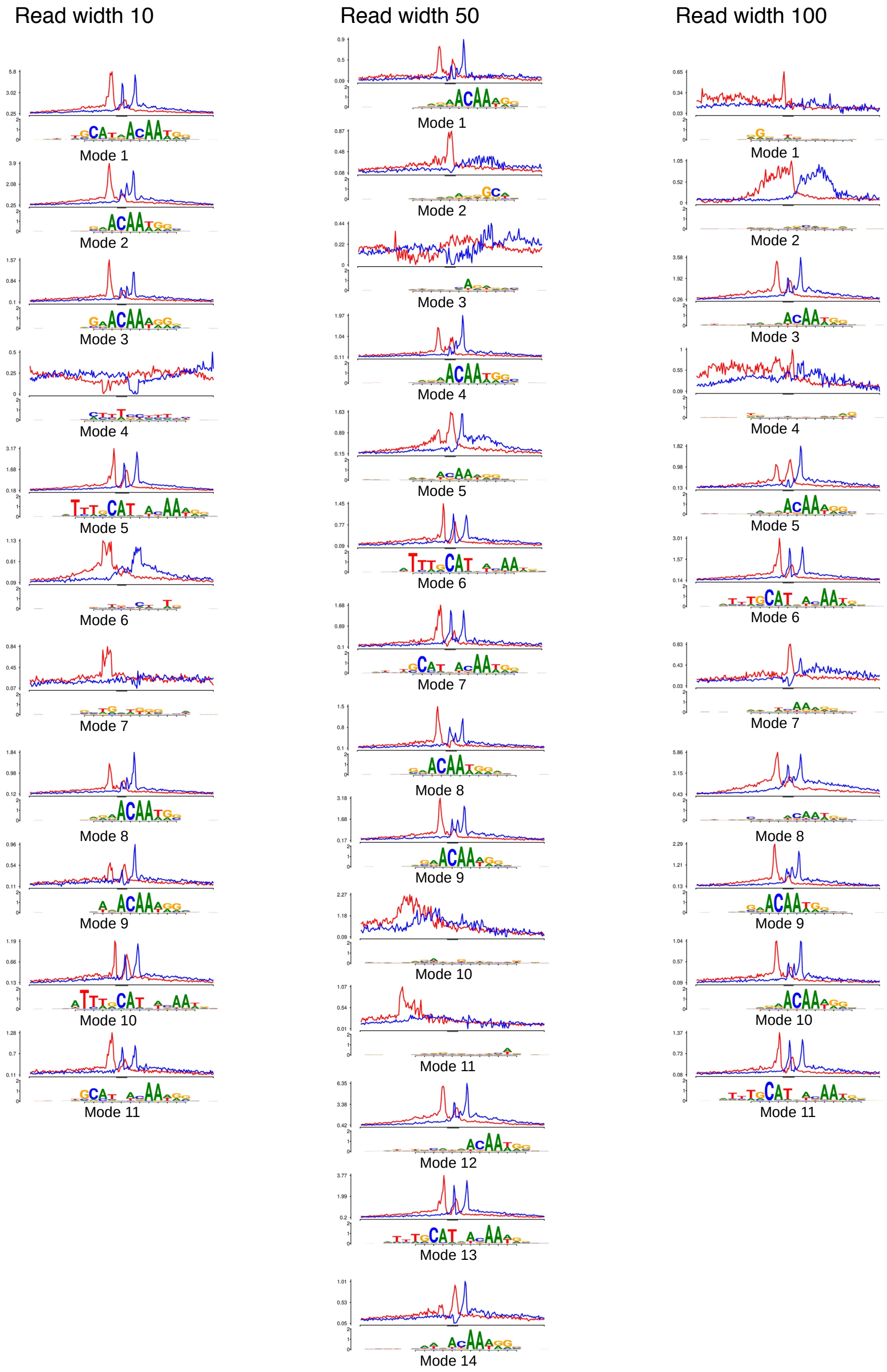

Fig. S8. ExoDiversity was run with three different values of read widths on the Sox2 dataset. For larger widths, motifs become distinctly weaker. The average read plots are computed over a 200bp neighborhood around the motif: the ticks correspond to the motif positions in that mode shown below the plot in each case.

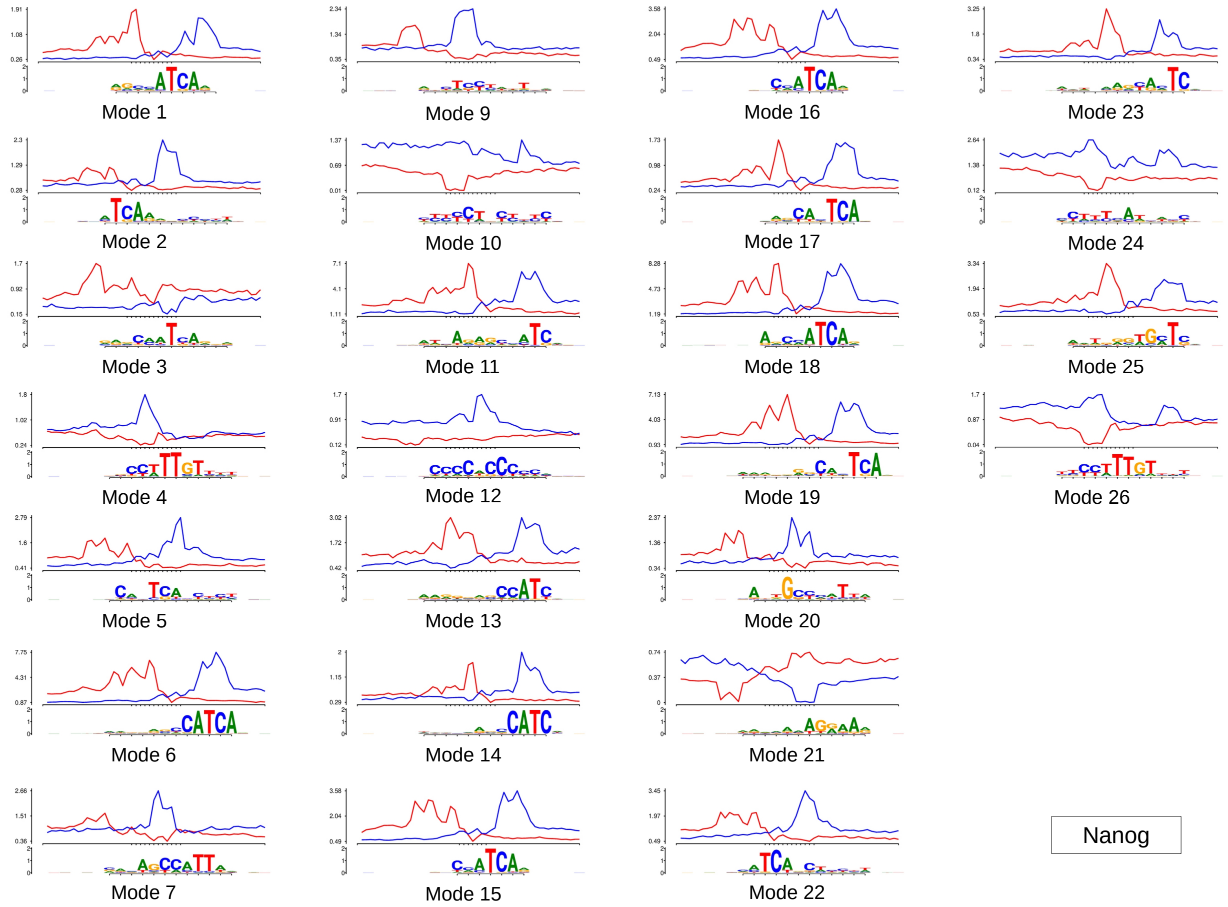

Fig. S9. The output on the Nanog dataset in mouse ESCs. Multiple variants of `ATCA` along with differing read distributions are detected. Sox2 motifs (modes 4, 21, 26) and Klf4 (mode 12) are also detected. The average read plots are computed over a 50bp neighborhood around the motif: the ticks correspond to the motif positions in that mode shown below the plot in each case.

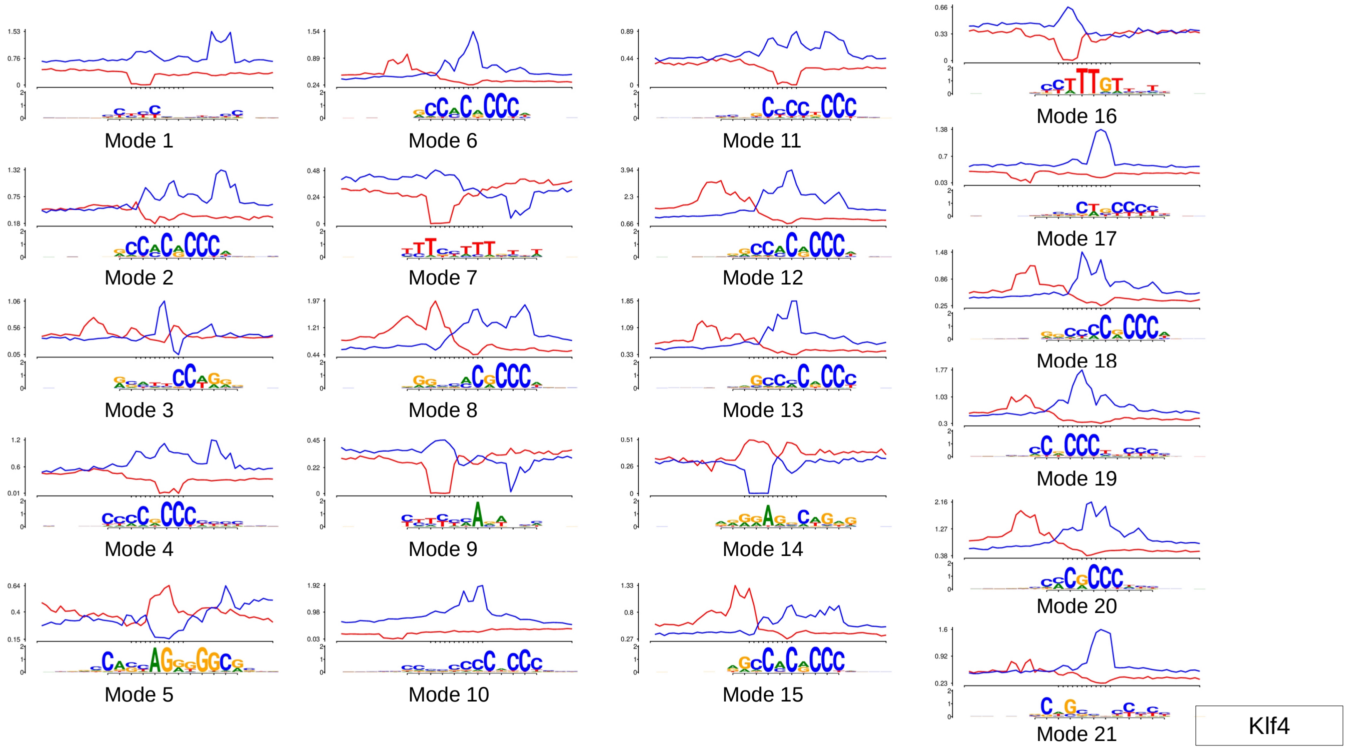

Fig. S10. The output on the Klf4 dataset in mouse ESCs. Multiple variants of the canonical Klf4 motif along with differing read distributions are detected. Additionally, Sox2 motifs (mode 16) and CTCF (mode 12) are also detected. The average read plots are computed over a 50bp neighborhood around the motif: the ticks correspond to the motif positions in that mode shown below the plot in each case.

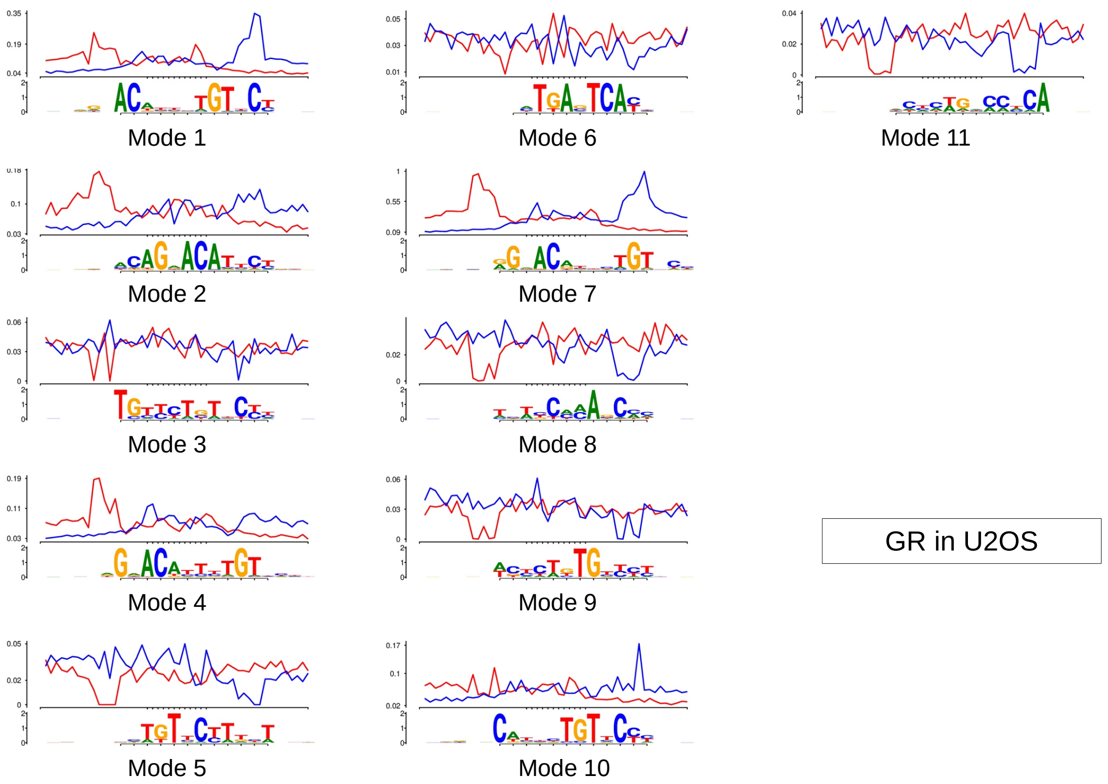

Fig. S11. The output on GR-bound regions in U2OS (osteosarcoma) cells. In addition to several GR motif variants, the AP1 motif (mode 6) is also detected.The average read plots are computed over a 50bp neighborhood around the motif: the ticks correspond to the motif positions in that mode shown below the plot in each case.

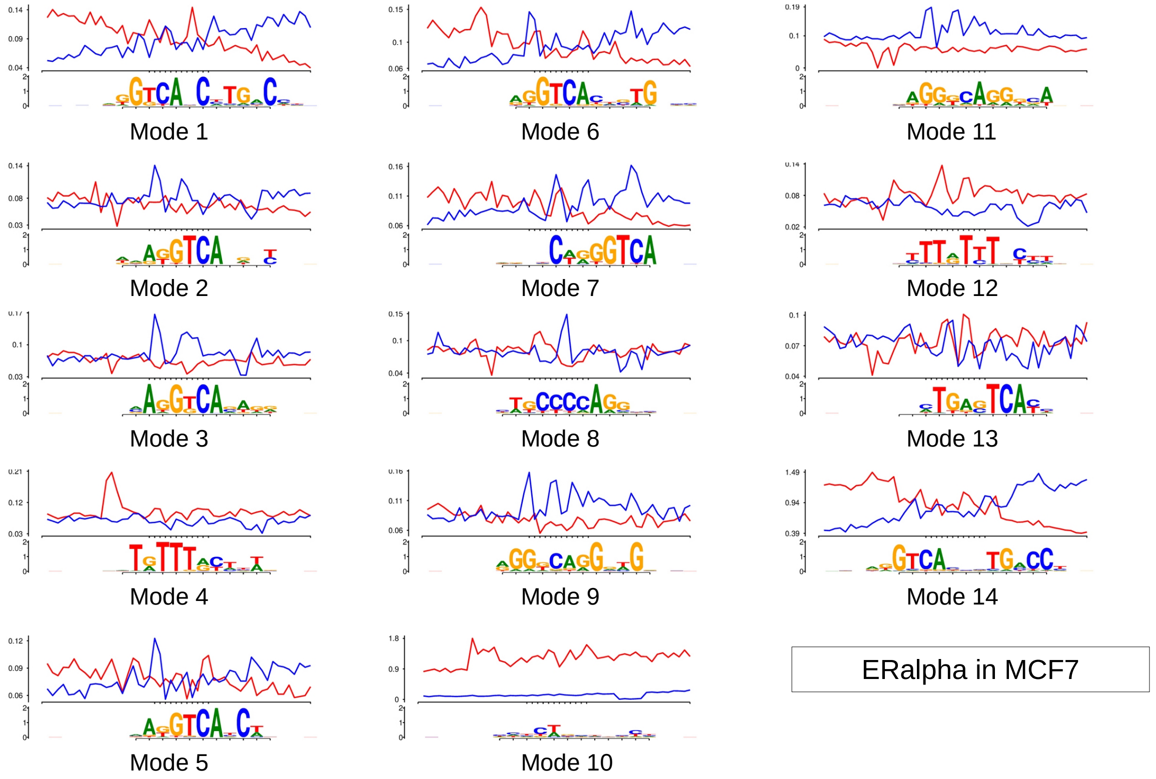

Fig. S12. The output on ERα-bound regions in MCF7 cells. In addition to the ERα motif variants, FoxA1 (mode 4) and AP1 (mode 13) are detected. The average read plots are computed over a 50bp neighborhood around the motif: the ticks correspond to the motif positions in that mode shown below the plot in each case.
